# ‘Free From Doubt’? The border between charity and the state in the British National Health Service, 1948–74

**DOI:** 10.1093/tcbh/hwag005

**Published:** 2026-04-14

**Authors:** Hannah Blythe, Martin Gorsky

**Affiliations:** Centre for History in Public Health, London School of Hygiene and Tropical Medicine (LSHTM), 15-17 Tavistock Place, London WC1H 9SH, United Kingdom; Centre for History in Public Health, London School of Hygiene and Tropical Medicine (LSHTM), 15-17 Tavistock Place, London WC1H 9SH, United Kingdom

**Keywords:** National Health Service, charity, hospital, welfare state

## Abstract

Britain’s National Health Service (NHS) is widely regarded as epitomizing statist healthcare. However, before its launch in 1948, Britain had a ‘mixed economy of welfare’ in which charity loomed large, most notably in the voluntary hospitals. This article examines the legacy of this charitable past in the NHS’s early decades. Although at its foundation, the Minister of Health, Aneurin Bevan, was determined that the NHS should end reliance on the ‘caprice of private charity’, political considerations meant that teaching hospitals retained their charitable wealth. Bevan’s policy was that the Exchequer would cover all ‘ordinary expenditure’, with voluntary reserves used only for special purposes, including research, additional comforts and amenities. Thus, a theoretical border between state-funded essentials and charitably financed inessentials was drawn for hospitals in the early welfare state. This article asks whether this border held true in practice. Its method is a comparative history of three case studies: St Bartholomew’s Hospital in London, the United Sheffield Hospitals, and the Royal Infirmary of Edinburgh. We present quantitative and qualitative analysis of endowment expenditure between 1948 and the NHS’s reorganization in 1974. Our findings demonstrate that expectations of a simple division between essential and non-essential aspects of healthcare proved naïve. Charitable spending notionally on research, amenities, and equipment was soon geared to clinical care. It also sustained capital programmes, disproportionately advantaging those hospitals with large inherited assets. Hospital charity also served as a rhetorical and visual device, promoting an ideal of humane care and legitimizing the place of voluntarism within the NHS.

## Introduction

It is a truism that health systems typically combine public, private, and non-profit forms of funding and provision, even though the precise mix may vary across time and place. Britain’s National Health Service (NHS) is widely regarded, at least in its early phase, as exemplifying the statist approach. Funding came principally from taxation, hospitals were mostly public institutions, primary care was contracted by the government, and entitlement to medicine was universal, free at the point of use. Before the Service’s launch in 1948, though, Britain had epitomized a classic ‘mixed economy of welfare’.^1^ A large private sector included general practice and nursing homes. Local taxes and the Poor Law funded psychiatric and general hospitals and the gamut of public health, while national health insurance covered lower earners for primary care and income support.^2^ Charity loomed large, especially in the historic voluntary hospitals. These dominated acute medicine, and were funded by philanthropy and small contributions, overseen by volunteer trustees, and staffed largely by honorary practitioners.^3^ The legacy of this charitable past in the era of the NHS hospital is our subject.

In presenting his NHS proposals to Parliament, Labour’s health minister, Aneurin Bevan, had argued charity was no longer appropriate in a modern health system. Its ‘caprice’ had produced spatial inequity in the distribution of resources. Bevan believed that charity’s paternalistic overtones alienated working-class users, and that it was ‘repugnant to a civilized community’ that clinical services should depend upon ‘nurses … going about the streets collecting money’. Nor did Bevan see its much-vaunted humanitarianism as relevant in an age of scientific medicine: better to be ‘kept alive in the efficient if cold altruism of a large hospital than expire in a gush of warm sympathy’.^4^ Bevan accepted, just as Beveridge did, that voluntary action ‘for social advance’ was part of the fabric of society and culture, but he now envisaged for it only a supplementary role, ‘where the shoe pinches’.^5^

However, implementing this transition was a policy challenge, for the British voluntary hospitals had deep roots in civic life.^6^ Overall, there were just under 1,500 by the late-1930s, accounting for about one-third of all beds, with the rest in the municipal sector, Poor Law, or county mental hospitals.^7^ They included major teaching and general hospitals in the urban centres, some with mid-eighteenth-century roots, along with small-town cottage hospitals and institutions for specialties like maternity care and children’s illnesses.^8^ Their charitable funding had blended subscriptions, donations, legacies, and earnings from property or accumulated investments, supplemented in the late-Victorian period by organized collections through the church or workplace. By the inter-war years, the latter had become a nationwide network of workers’ contributory schemes, a mutualist form which gave subscribers a notional right to care.^9^

Bevan’s problem, then, was how to deal with the endowments of the major voluntary hospitals, now that these were to be ‘nationalized’ under the NHS. Treasury estimates in the early 1950s (current prices) put the English and Welsh hospitals’ endowed wealth at about £40 million, with £13 million the figure for Scotland.^10^ Should Bevan, like Henry VIII, dissolving the monasteries, simply absorb this into the Exchequer? Or should he permit charity’s continuance while more strictly circumscribing its uses? He chose the latter solution in the NHS Acts (1946–7).^11^ In England and Wales, to placate elite medical opinion, the teaching hospitals remained independent with their own Boards of Governors (BoGs), and kept their endowments. The other voluntary hospitals pooled theirs in a Hospitals Endowment Fund, to be distributed between fourteen new Regional Hospital Boards (RHBs), into which most institutions were grouped. In Scotland, teaching hospitals were placed with the others under five RHBs, and a Hospitals Endowment Commission (HEC) oversaw disbursement.^12^ There would be a clear delineation—a border—between the roles of the state and charity. Henceforth, the Treasury would cover all ‘ … ordinary expenditure … including capital expenditure’.^13^ Charity would fund ‘research as the Board think fit’ and the vaguely specified ‘purposes relating to hospital services … ’, which were in practice understood as ‘amenities’ for staff and patients.^14^ A Ministry of Health circular in 1948 clarified that hospitals now were ‘in no way dependent on voluntary financial help for their normal needs’, and to counter any such ‘false impression’, RHBs were prohibited from ‘appealing for or collecting funds’. All were now ‘free from doubt’ as to where the border with charity’s responsibilities lay.^15^

The aim of this article is to establish whether this notional border held good in practice. Our focus is narrow, on hospitals’ endowed charitable wealth, and does not range more widely into different forms of health voluntarism, nor into the primary or social care sectors. We interrogate the underlying conception of charity’s place in the new welfare state implied by this distinction between core and ‘extra’ services. Chronologically, our focus is the era of the Bevanite settlement, between the NHS’s launch in 1948 and the structural reforms of 1974, before the lifting of the ban on hospital charity fundraising in 1980. Because national-level data do not permit a clear answer to the question, our method here is a fine-grained analysis of three case-study hospital groups, in London, Edinburgh, and Sheffield.

We begin with a fuller historiographical discussion, situating our enquiry within recent literature on voluntarism and the NHS. We then introduce our case studies, describing data and methods. The central sections are mainly empirical, first presenting overall spending patterns, then examining details and representations of the main sub-categories: research, medical and surgical equipment, amenities, and capital works. We show that research received considerable charitable money from the outset, but, over time, the border between research and patient care emerged as a site of negotiation. Medical and surgical items deemed routine received endowment funds, with managers at all three hospitals feeling increasingly obliged to compensate for Treasury stringency to keep pace with drugs and equipment costs. Amenities and comforts were initially designated obvious domains for endowments, but this relatively small use of charity became an outsized public signifier that NHS teaching hospitals had the capacity to look after well-being as well as health. What is more, the Boards’ varying charitable resources led to differing judgments regarding what constituted ‘essential’ provisions. Finally, although the managers of our teaching hospitals saw capital investment as the Exchequer’s responsibility, the Boards quickly began to use their endowments to expand facilities and modernize infrastructure as Treasury stringency became apparent. Our analysis shows that the integrity of the theoretical border did not translate into reality, but that it nonetheless remained as a rhetorical device that hospital managers harnessed to lobby for greater funding. Our conclusion discusses the generalisability of these findings and reflects on their larger implications for understanding the place of charity in a universalist welfare state.

## Histories of charity and health voluntarism

Until the 1980s, the history of British social policy was dominated by a ‘welfare state escalator’ narrative, premised on an inexorable ascent towards greater public provision. Then, as a reconfiguration of Britain’s welfare system began in the Thatcher years, new studies of charity and voluntarism emerged, alongside re-evaluations of their pasts.^16^ Resultant intellectual histories of charity depicted a mid-twentieth-century reorientation, when a ‘language of sectors’ arrived—public, private, and voluntary—through which economists and social administrators reconceptualized the delivery of social goods.^17^ Delineating these sectors allowed the different attributes of each to be theorized. In the health arena, the concept of ‘market failures’ like information asymmetries (thanks to doctors’ greater technical knowledge) and catastrophic costs (when even prudent savers could not afford medical expenses) helped explain why medicine could not be a purely commercial transaction. Meanwhile, ‘voluntary failures’ like those identified by Bevan—inequity, paternalism, inefficiency—explained why charity alone could not provide the alternative.^18^ Nonetheless, contemporaries argued that charity still merited a place in the welfare state, fulfilling roles that government could not, such as mobilizing community engagement, representing marginal groups, and pioneering novel areas of concern. Historians appropriated the attendant notion of a ‘moving frontier’ between voluntarism and the state to capture this changed dynamic.^19^

Meanwhile, studies of the pre-NHS hospital concentrated on the viability of a system reliant on charity. Some, in the wake of Titmuss’s early reading from 1950, stressed that voluntary healthcare had become vulnerable as demand for institutional biomedicine had escalated, with rising expenditures and diminution of capital assets.^20^ These commentators note too that the increasingly formalized contributory schemes were displacing traditional donations and subscriptions as a funding source, firstly because income tax and death duties had discouraged philanthropic giving, and secondly because the expansion of municipal, tax-funded medicine instilled notions of hospital care as a right.^21^ Later readings added nuance to this reductive picture of voluntary failure, with urban case studies highlighting individual places with thriving income streams. Grounded in the affective bonds of surrounding communities, these local healthcare systems were sustained by civic philanthropy and mutualism.^22^

Such reappraisals raised the idea of continuities across the purported rupture of the post-war moment. The consensus is that health voluntarism remained, but in a transformed state, now flourishing principally in the work of associations, non-governmental organizations (NGOs), and social movements outside the NHS.^23^ Concerned either with diseases or pathogenic behaviours, this activity functioned through advocacy groups, loci of expertise, and user networks.^24^ Within the NHS itself, active charity was small scale. For example, the Rockefeller Foundation’s support for innovative British health centres briefly continued this American philanthropic body’s interwar promotion of social medicine.^25^ Other studies detail path-dependent survivals of community action in NHS hospitals, for example, by the Women’s Voluntary Service, Hospital Sunday adherents, and the Red Cross, and show how Leagues of Friends operationalized the Bevanite framework by providing ‘extras’ like tea shops and televisions.^26^ Investigations of activism show that, from the 1970s, voluntary engagement included defending NHS institutions against cuts.^27^ These studies also begin to illuminate the subjectivities of people who worked in and used hospitals, an area explored more fully by visual and material historians of the NHS. Such work examines how architecture, ward design, and colour schemes shaped the experience of hospital users, and mediated political loyalties towards the Service.^28^ The question of whether charity contributed to these cultural constructs remains unexamined.

There is only a limited historiography with respect to our main theme, hospital charities under the early NHS. Harris and Cresswell outline the administrative structures and accompanying official guidance for hospital endowments, arguing that the boundary between essential and non-essential purposes was vague from the start. Their national-level data on endowment expenditure trends points to areas like ‘hospital purposes’ and capital expenditure, where it was likely substitutive, not supplementary, to core NHS activities.^29^ Meakin covers the legal situation.^30^ Scholars generally skim past the post-war decades, viewing Margaret Thatcher’s election as the moment when NHS voluntarism became significant. It is true that the rise of Thatcherism and the attendant lifting of the ban on NHS charity fundraising in 1980 had an impact. The simultaneous retrenchment of the welfare state coincided with the take-off of high-profile NHS hospital charity appeals led by professional fundraisers. This format was pioneered by the 1987–88 ‘Wishing Well’ campaign for Great Ormond Street Hospital (GOSH) for Sick Children.^31^ Quantitative data confirm an overall increase in voluntary income and expenditure in NHS hospitals in this period.^32^ Research focused on London has also suggested that the ‘border’ began to be breached in the 1980s by large teaching hospitals using charitable wealth to preserve their position in response to proposals to reform resource allocation methods and divert funds away from the capital.^33^

Recent contemporary studies with a broad geographical scope have drawn two key conclusions about the allocation and distribution of charitable wealth in the NHS in the present and recent past. First, these investigations indicate that an emphasis on amenities and comforts remains, but voluntary spending on areas like education and equipment now substitutes for Exchequer funds. Second, voluntary spending capacity varies drastically between geographical locations and different types of hospitals. For example, children’s hospitals have much greater charitable resources than psychiatric hospitals.^34^ The debate over whether charity should have a role in core NHS services was reanimated in the 2020s by the COVID crisis and the much-publicized philanthropy mobilized by ‘Captain Tom’.^35^

We contribute to this debate by helping to establish the historical role of voluntarism in the NHS. Our main contribution will be to the question of whether the Bevanite delineation of charity’s new, lesser role obtained under the early NHS. This will firstly complement Harris and Cresswell’s findings, testing their propositions with evidence from the local level. Secondly, by looking beyond active voluntarism, we will add another dimension to accounts of the voluntary sector under the welfare state, and the question of continuities across the start of the NHS. Finally, in examining the uses and representations of hospitals’ charity, we will explore connections with cultural histories of the Service, reflecting on whether voluntarism acted as a vehicle for mobilizing sentiment of NHS users.

## Sources and methods

We use case studies from London, Sheffield, and Edinburgh to assess how national policies operated in three distinctive parts of the UK. The concentration of charitable resources in the metropolis makes one London-focused case essential, and St Bartholomew’s (Bart’s) exemplifies a wealthy teaching hospital. By 1946, its endowment was generating amongst the highest income from charitable investments per bed in the UK, and its location placed Bart’s within a nexus of prestigious philanthropic hospitals associated with medical innovation. For contrast, we also investigate the United Sheffield Hospitals (USH), a teaching hospital serving a large industrial city in northern England, functioning like Bart’s under the stipulations regarding England and Wales in the NHS Act (1946).^36^ We contrast our English examples to the Royal Infirmary of Edinburgh and Associated Hospitals (RIE), to establish how the distinctive endowments policies of the NHS (Scotland) Act (1947) worked. Its position as Scotland’s largest former-voluntary hospital, situated within its wealthiest RHB, provides a useful comparator.^37^

Bart’s began as a medieval religious institution for healing and care of the sick. The hospital was founded in 1123 as part of the Priory of St Bartholomew, and continued functioning after the dissolution of the monasteries, remaining a voluntary hospital until 1948.^38^ During our period, two further institutions came under the auspices of Bart’s Governors: the Alexandra Hospital for Children with Hip Disease, based in Luton from 1940 and managed as a unit of Bart’s until closure in 1958; and Hill End Mental Hospital in St Albans, to which Bart’s staff and patients had been evacuated in the Second World War, and where some activities remained until 1961.^39^ Bart’s was located within the boundaries of the North East Metropolitan RHB (NEMRHB), one of the four RHBs covering the populous city of London and its concentration of hospitals.^40^

The RIE was founded in 1729 as one of the earliest eighteenth-century voluntary hospitals.^41^ By 1958, the nationalized RIE included: Edinburgh’s Royal Infirmary, Simpson Memorial Maternity Pavilion, Queen Mary Maternity Home, Convalescent House at Corstorphine, Beechmount Hospital (another convalescent facility), and Edinburgh Dental Hospital and School.^42^ The RIE group operated under a Board of Management (BoM), administered within Scotland’s wealthiest RHB, the South-Eastern RHB (SERHB). Scotland’s other RHBs, in descending order of charitable income generated per bed were: North Eastern (covering Aberdeen, alongside the Shetland and Orkney Islands); Eastern (containing Dundee); Western (grouping Glasgow with the Argyll Islands), and Northern (the largest settlement in which was Inverness in the Highlands, which were administered alongside Skye and the Outer Hebrides).^43^

The Sheffield institutions were more recent. The USH included the Royal Infirmary and the Royal Hospital (general hospitals founded in 1797 and 1834, respectively), alongside the Jessop Hospital for Women, Sheffield Hospital for Children, and Edgar Allen Physiotherapy Centre.^44^ The USH was formed in 1948 when the city’s former-voluntary teaching hospitals were nationalized and united under a BoG, and sat within the geographical limits of the Sheffield RHB (SRHB), which also covered much of Derbyshire, Nottinghamshire, Leicestershire, and Lincolnshire.^45^ The USH was abolished with the 1974 NHS reorganization, and, soon after, the Royal Infirmary and Royal Hospital’s services were transferred to the new Royal Hallamshire Hospital, built by the BoG.^46^

We conducted quantitative analysis of these hospitals’ charitable income and expenditure to establish overall patterns of activity and to determine how classification related to the ‘border’. To account for inflation, we adjusted financial time series to constant prices (1949), and where comparative figures are mentioned in the expository text, these are at 1949 prices unless otherwise stated. We supplemented these investigations with qualitative analysis of administrative records to gauge the extent to which border imperatives shaped decision-making. Each group of managers had a different attitude to accounting and public communications, meaning that the materials available for each case vary. We have, though, extracted comparable information from these diverse sources.

One source was the annual report, a mainstay of pre-1948 charity, used to publicize voluntary organizations’ activities, petition support for charities’ general causes, and fundraise.^47^ This was amongst the former-voluntary hospitals’ ‘worthy traditions’ which the USH’s Governors pledged to maintain. Their first report declared that the new ‘service should command not only the sympathy and support of certain individuals but also the sustained interest of the general public’.^48^ Reports were then disseminated to earn support for the USH and to provide the local population with opportunities to engage in hospital life. A full set of publications for 1948/49–1973/74 survives, chronicling hospital activities and containing detailed financial accounts. While a report exists for every year, changes in accounting methods mean that we have comparable data for the financial years 1950/51–1972/73.

The RIE’s BoM also valued annual reports, but initially ceased production under the NHS. Management reprised the tradition in 1958, publishing an inaugural report to cover the first decade of NHS activity, though without retrospective accounts.^49^ We, therefore, have a full set of RIE accounts from 1957/58 to 1972/73, alongside qualitative information and data from accompanying public documents.

By contrast, Bart’s leaders ceased publishing annual reports at the outbreak of the Second World War, and did not reinstate the practice.^50^ We instead rely on Bart’s internal administrative records. A set of accounts covering Exchequer and total endowments income and expenditure, 1950/51–1972/73, exists, though occasional years are missing.^51^ The archive also contains the complete Discretionary Fund (DF) accounts for every year, 1949/50–1968/69, incorporating most of Bart’s endowments, but excluding several minor special funds.^52^ We use the DF’s detailed accounts for the majority of our analysis, and supplement our figures with information about the small separate funds and data from the incomplete accounts. We complement these figures with qualitative records regarding spending decisions, using minutes of the executive, finance, and research committees.^53^

## Pattern of overall expenditure

The Government’s announcement that the Exchequer would finance all core NHS functions created a notional border between essential services and additional improvements while permitting ongoing voluntarism. All three hospitals’ boards seized this opportunity to continue using charitable money, but they held very different levels of voluntary resources, and the RIE was affected by Scotland’s NHS settlement. Overall trends in charitable expenditure consequently varied significantly.

Our three Boards held their charitable wealth, in different proportions, in investments and securities, land, property, and cash. Income had originally been derived from estates, interest on investments, legacies, subscriptions, and donations from individuals and organizations. Cumulative asset bases and the aforementioned ‘caprice’ of gifting had led to much prior variability. While we lack data on capital accounts before the NHS, this variability is simply demonstrated through differential levels of total voluntary income supplied by charity, contributory schemes, and interest yields. For example, in 1938, before wartime disruption, hospitals in the Bart’s group received £243 per bed, but the figures for the Sheffield and Edinburgh groups were £204 and £168 respectively (current prices).^54^

Inequities were carried over into the NHS era, with English teaching hospitals retaining their whole endowments. In 1949, the USH’s Governors held an endowment of £490,075, which equated to £354 per bed.^55^ In 1951, Bart’s endowments totalled £2,725,296, amounting to the far more substantial £4,439 per bed. Between these poles were the RIE’s voluntary funds, whose market value in 1948 had been £1,324,128 (current prices), but which, a few years after Scotland’s first redistribution was equivalent to £856 per bed by 1957/58.^56^

Governance arrangements varied too. Of Bart’s endowment, the majority was overseen by the BoG in its DF, though several special funds, amounting in 1951 to about seven per cent of the whole, were managed by a separate Voluntary Board. These included, for example, allocations from the pan-city Metropolitan Hospital Sunday Fund, created in 1873 to collect donations for poor Londoners needing medical treatment.^57^ Only sporadic records of these special funds survive, making aggregation of this small percentage of Bart’s charitable monies impossible, hence our reliance on the DF only in subsequent analysis. While the USH was undoubtedly the least wealthy at the NHS’s inception, another pot of money became associated with the hospital in 1953. The Million Pound Appeal (MPA) had been launched in 1938 by leaders of the Royal Hospital and Royal Infirmary. This had separate accounts and governance arrangements and was ringfenced for building a new general hospital, though if included in a notional calculation of the USH’s charitable wealth, this would have been raised to £752 per bed in 1953.^58^ This increase was not entirely due to the MPA, but was also because the value of the USH’s endowment had recently grown considerably (it peaked in value in 1952).

How were hospitals affected by the NHS Act (Scotland), under which the HEC was to redistribute charitable resources? In the mid-1950s, the HEC determined that the majority of endowments should be redistributed across the five newly established RHBs, four of which then contributed to a new Scottish Hospitals Endowment Research Trust (SHERT), national allocations of which would facilitate research.^59^ A HEC memorandum to the RIE in 1954 directed Managers to transfer around one quarter of their assets to the West Lothian (Bangour) Hospitals, Edinburgh Royal Victoria and Associated Hospitals, SERHB, and SHERT.^60^ Redistribution was therefore substantial, but the RIE kept a considerable sum. In 1957/58, the Infirmary’s endowment was worth £652,355 (1949 prices), about half its value in 1948. Nonetheless, the RIE’s advantage over other SERHB hospitals remained. In 1955, for example, the RIE’s charitable income per bed was 7.5 times that of the Gogarburn Mental Deficiency Institution, the hospital with the lowest such income in the region. However, this still represented a step change, since none of the SERHB’s six hospitals for mental illness or what was then known as ‘mental deficiency’ had any recorded charitable income before the HEC’s redistribution.^61^ Disparity in voluntary income between teaching and psychiatric hospitals has been longstanding and geographically widespread, with similar inequalities evident in English NHS trusts between 2000 and 2021.^62^ Moreover, endowments by this point only constituted just over half of the RIE’s charitable assets. The Board kept all of the ‘legacies, donations, etc’ received since the ‘appointed day’ in a separate BoM Fund, which along with the endowments, totalled £856 per bed.^63^ The contrast with the most prosperous English hospitals persisted and the RIE’s Managers certainly lamented their comparative disadvantage.^64^

Scottish legislation meant that no BoM could be sure of keeping their retained endowments, and the RIE faced later reduction of charitable funds. In 1968, the Board criticized the Secretary of State for Scotland’s review of the hospital endowments scheme. Managers did not openly oppose efforts to lessen resource inequalities, but warned that:

In England the teaching hospitals have not had to bear any reduction and a further reduction of the funds of the Scottish teaching hospitals can only widen, to the detriment of Scottish teaching hospitals, the gap which already exists between them and the English teaching hospitals.^65^

Nonetheless, the Board’s endowment was reduced in 1972 to just £8,615.^66^ Historical precedent permitted the RIE Managers to retain substantial assets in their BoM Fund. However, by way of comparison, having entered the NHS with a wealth base 2.7 times the size of USH’s, by the 1970s, the RIE’s voluntary assets were now less than those of the Sheffield hospital due to redistribution within Scotland.^67^

How did the managers of the charitable funds initially understand the new dispensation? At the NHS’s birth, the English Boards of Governors affirmed their adherence to the Bevanite border, while acknowledging that legislation permitted flexibility. The USH’s Board remarked in 1949 that endowments ‘may be used to provide, among other objectives, amenities for patients and staff, and to promote medical and other research’.^68^ The phrase ‘other objectives’ was vague and allowed for discretion. Bart’s Executive Committee minutes that year similarly described charitable ‘monies for additional amenities to patients and staff, for research and other appropriate purposes’.^69^

The Scottish HEC offered to the RIE management differing guidance regarding endowment expenditure than it did to the RHB. Its 1954 memorandum articulated expectations regarding the SERHB’s charitable spending:

The fund … at the disposal of the Regional Hospital Board is intended to meet expenditure within the Region which could not reasonably be met by Exchequer sources or from the funds of any one Board of Management, including, for example, expenditure on Regional conferences, staff training … .^70^

The Commission had also considered how far it should intervene in the RIE BoM’s activities, and wondered if ‘the purposes for which Endowments might be used should be specified in detail or whether only a general direction should be given to the Board’. The HEC ultimately decided upon the latter, counselling ‘that the funds be used for such purposes relating to the hospital or specialist services or to the functions of the Board with respect to research as the Board may see fit’.^71^ This guidance also indicated greater freedom of charitable expenditure for the RIE’s Board than the English Governors envisaged for themselves. Latitude in decision-making was present from the start.

**Figure 1:**
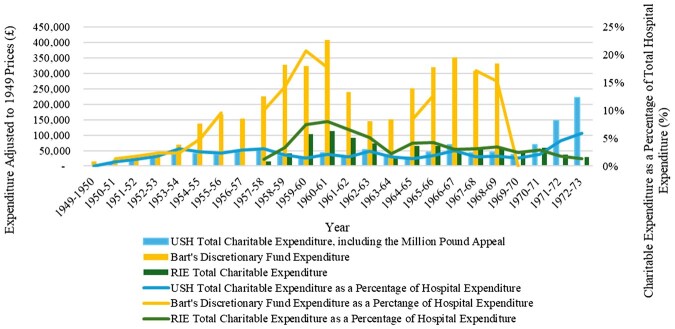
Charitable expenditure, Bart’s, Sheffield and Edinburgh hospitals, 1949/50–1972/73, at constant prices (1949).

Presenting the overall patterns of charitable expenditure in the case study hospitals allows examination of how these expectations translated into practice. Figure 1 shows the trends at Bart’s (DF), the USH (endowments plus MPA), and the RIE (endowments plus BoM Fund) over time. Values are deflated to 1949 prices. The bars show annual amounts of expenditure, and the lines represent these sums as percentages of all (charitable plus non-charitable) hospital spending. The breaks in the Bart’s line series result from missing data.

For most of the period, Bart’s charitable spending far exceeded that of the other hospitals, peaking in 1961/62 at £409,390 and fluctuating considerably. This outlay also generally constituted a much greater proportion of overall expenditure than at the USH and RIE, with the DF providing 21 per cent of Bart’s overall outgoings in 1959/60. The USH’s voluntary spending instead remained consistently quite low, both in terms of amount and proportion of overall outgoings, which mostly hovered between 1 and 3 per cent. However, in 1971/72 and 1972/73, the Governors used the MPA money, raising total charitable spending to £146,631, then £221,407, 5 and 6 per cent of total hospital expenditure respectively. The RIE’s charitable outgoings were more similar to the USH’s, especially throughout the final decade, with a peak during the early 1960s. However, the graph under-represents actual charitable expenditure at the RIE because research allocations from the SHERT were not recorded in the hospital’s accounts.

**Figure 2:**
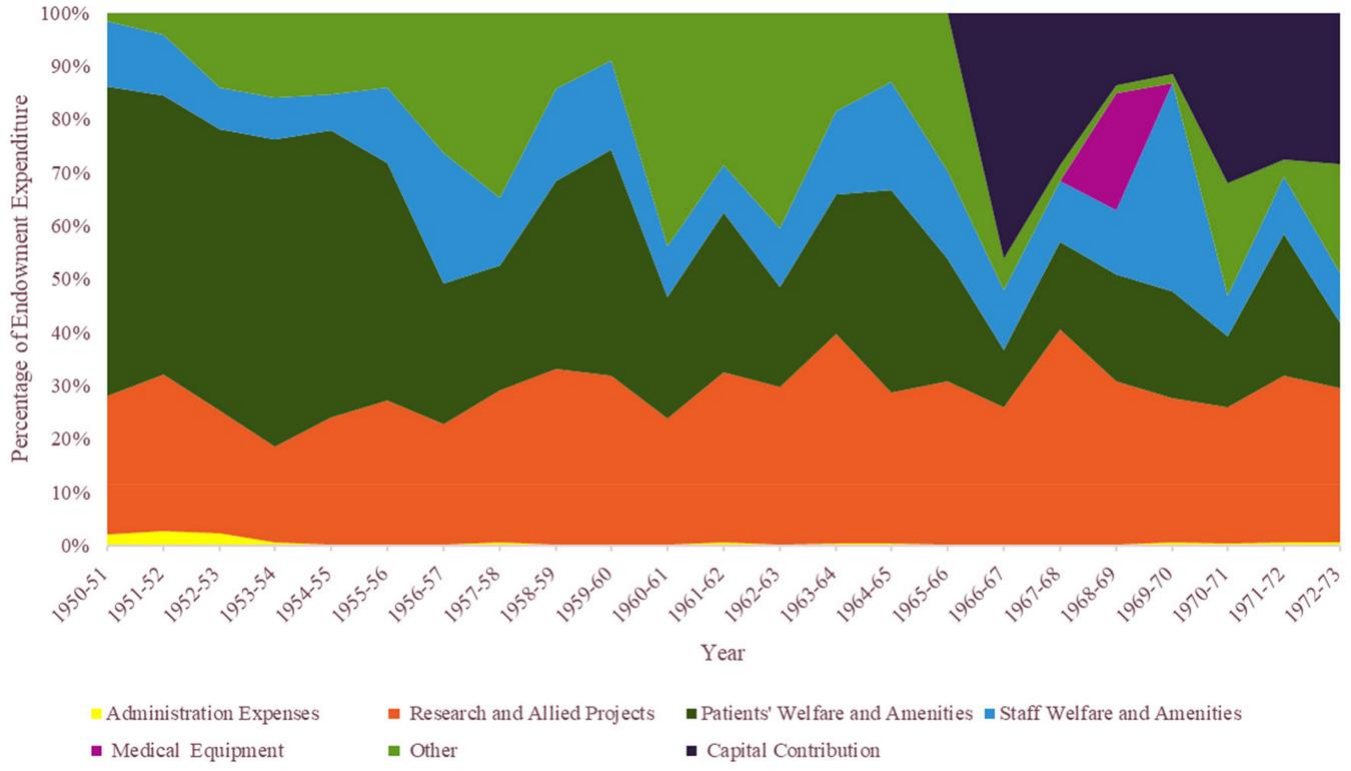
USH charitable expenditure by category, 1950/51–1972/3.

**Figure 3:**
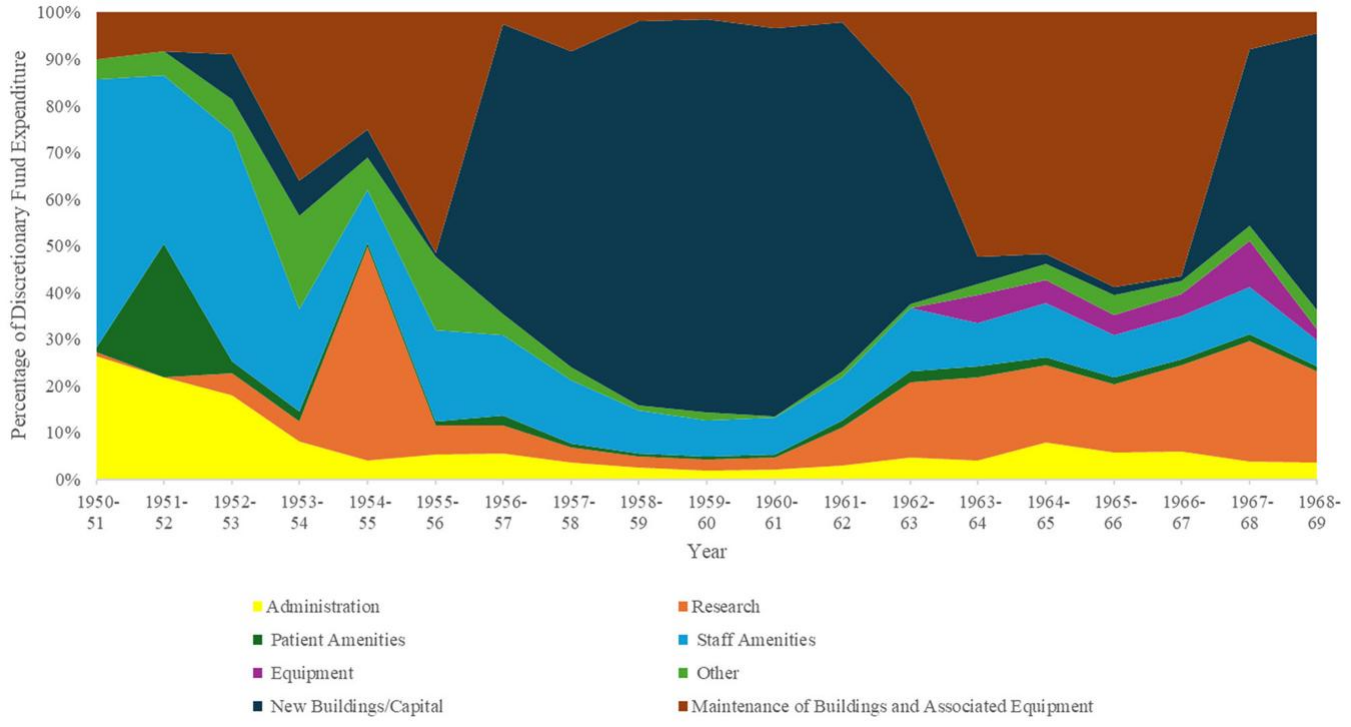
Bart’s charitable expenditure by category 1950/51–1968/69.

Disaggregating each hospital’s charitable expenditure illuminates their differing spending patterns, revealing shifting and varying usage on research, capital, and routine investment in buildings and equipment, and patient and staff amenities and comforts. The USH endowment fund and Bart’s DF accounts offer detailed expenditure breakdowns, from which we have plotted colour-coded graphs (Figs 2 and 3) using broadly comparable spending categories. The RIE accounts contain less comprehensive analysis, so we present the Edinburgh data separately (Table 1). Some of the USH and Bart’s spending categories are almost directly comparable. The USH accounts list ‘Research and Allied Projects’, ‘Staff Welfare and Amenities’, and ‘Patient Welfare and Amenities’; while Bart’s include ‘Research’; ‘Amenities to Patients’; and ‘Amenities to Staff’. Bart’s category of ‘New Buildings and Equipment’ is a broad proxy for the USH’s ‘Capital Contribution’. However, the USH’s accounts do not contain a classification comparable to Bart’s ‘Maintenance of Buildings and Associated Equipment’. The graphs reveal that research consistently attracted a significant proportion of the USH’s endowment expenditure, while patient welfare and amenities occupied a declining percentage of outgoings. At Bart’s, expenditure on new buildings and equipment dominated discretionary spending at certain points, but received a much smaller fraction of outgoings when allocations to buildings and equipment maintenance were high. Table 1 presents the less detailed breakdown offered in the RIE’s accounts, showing that ‘Alterations, Furniture and Equipment’ consistently received the greatest proportion of the group’s charitable outlay, ranging between 69 and 92 per cent.

**Table 1:** Charitable expenditure by category, Royal Infirmary of Edinburgh, 1957/58–1972/73.

Year	Research expenditure		Miscellaneous, incl. amenities		Alterations, furniture, and equipment		Administration		Total (endowment + BoM Fund)
	£	%	£	%	£	%	£	%	£
1957–58	1,303	9	2,002	14	10,984	75	271	2	14,561
1958–59	2,493	6	2,978	7	35,998	86	221	1	41,689
1959–60	2,226	2	11,187	11	89,892	86	998	1	104,303
1960–61	4,804	4	8,617	8	98,694	87	912	1	113,028
1961–62	3,178	3	4,937	5	83,018	91	263	0	91,394
1962–63	1,008	1	4,870	7	66,682	92	233	0	72,793
1963–64	2,270	7	4,390	14	25,085	79	151	0	31,896
1964–65	1,506	2	3,890	6	58,941	91	£157	0	64,493
1965–66	2,751	4	4,495	7	57,892	88	£328	1	65,468
1966–67	1,988	4	4,509	9	41,567	86	£184	0	48,248
1967–68	1,734	3	5,891	11	45,110	85	£180	0	52,915
1968–69	2,534	4	5,805	10	51,386	86	£148	0	59,873
1969–70	2,474	5	8,179	18	34,186	76	£153	0	44,991
1970–71	2,868	5	7,166	12	49,695	83	£130	0	59,860
1971–72	2,117	6	9,080	24	25,867	69	£209	1	37,273
1972–73	1,770	6	5,062	17	22,119	76	£191	1	29,143

Values deflated to 1949 prices.

**Figure 4:**
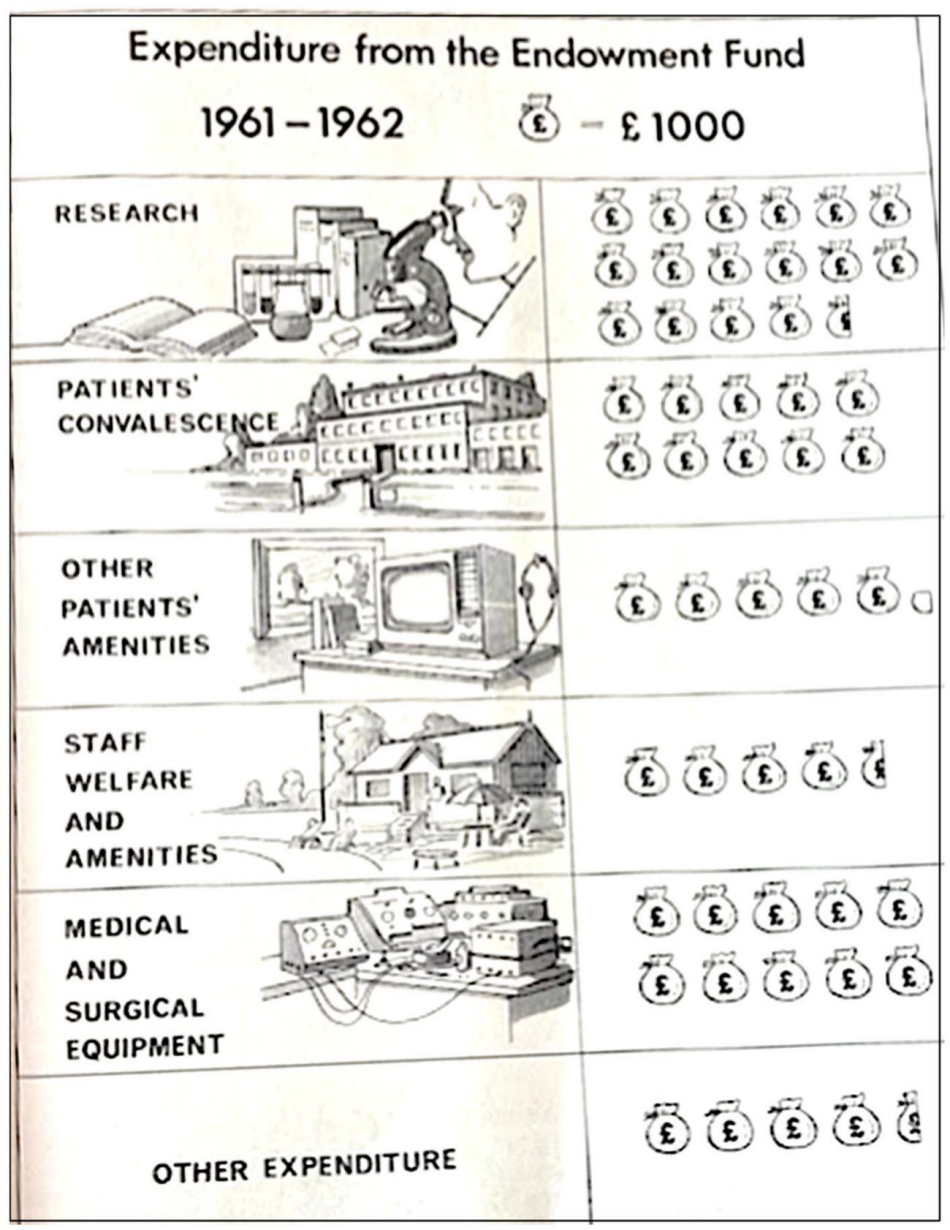
‘Expenditure from the endowment fund’, *USH AR*, 1962:17. Sheffield City Archives NHS28/1/2/1/3

These spending choices were marshalled in aid of NHS hospitals’ public images as well as their finances. Figure 4 is a representation from Sheffield’s 1962 annual report, which made much of the scientific modernity that charity underpinned, with illustrations of research and high-tech equipment sandwiching the infographic. A palatial convalescent home indicates institutional capacity and thereby the security on which NHS patients could rely, anchored in earlier traditions of civic voluntarism. Amenities for patients similarly depict novelty, with television and headphones, alongside comfortingly familiar books and artwork, while staff welfare is denoted by reclining figures on sun-loungers in a rustic setting. These signifiers of care, homeliness and warmth, coupled with advanced medical expertise, captured an ideal of the social democratic health system with state and voluntary sector in harmony.

In reality, the border between the remits of Treasury and charity quickly disintegrated, even as financial decisions continued to be made and justified in reference to a hazy and permeable divide between them. Our ensuing examination of the different expenditure categories demonstrates how this transpired, and our concurrent analysis of annual reports and committee minutes illuminates the reasons managers articulated for their spending decisions.

## Expenditure categories: research

Despite ‘the two main purposes of a teaching hospital’ being, as the 1948/49 USH report noted, ‘to treat the sick and to provide facilities for medical teaching and research’, the latter was not initially seen as part of the state-funded core medical service.^72^ Pursuing medical knowledge and prestige was framed as an additional activity. Bevan justified allowing teaching hospitals to retain endowments on the basis that these were ‘distinguished, to a very large extent, from the endowments of general hospitals because … [they] are earmarked for special purposes, such as cancer research’.^73^ The managers of all three hospital groups thus spent charitable money on research from the outset. However, over time, the border between research and patient care emerged as a site of negotiation. Drawing a line between research and cutting-edge treatment became increasingly difficult.

All three hospitals boasted diverse research regimes. The English hospitals derived most of their research budgets from their endowments, but both began to receive annual £10,000 (current values) Government research grants in the mid-1960s, representing a degree of re-evaluation of the Bevanite border at the state level.^74^ The RIE’s research programme was financed through a wider variety of sources. The BoM provided no systematic breakdown of their combined research expenditure, though annual reports sporadically mentioned the diversity of its research funders, including government bodies (Medical Research Council; Scottish Home and Health Department); non-governmental organizations like the Wellcome Trust; independent charities; the University of Edinburgh; pharmaceuticals companies; and, finally, the hospital’s endowment and BoM funds, SERHB endowments and the SHERT.^75^

The USH’s reports provide a granular breakdown of every endowment-funded project. Larger investments included £17,649 between 1951/52 and 1968/69 on investigating bronchitis, and £15,768 between 1964/65 and 1972/73 on neurological research. During the mid-1960s, Bart’s managers began to designate a small number of their schemes ‘Major Research Projects’. For example, between 1963/64 and 1968/69, psychopharmacology received £28,598, and metabolic and isotope research £52,307. The RIE’s programme was as broad as its funding sources, with research being woven into each department’s culture. In 1959, the RHB alone agreed to finance investigations into: radioactive isotopes, magnesium metabolism, clinical neuro-physiology, cardiopulmonary bypass apparatus, post-operative respiratory functions, hepatic blood flow, action of sulphonylureas and insulin, chronic lung disease, and renal disease.^76^

As administrators implemented projects, they came to see the work as occupying both sides of the border. Indeed, Bart’s flagship research project generated challenging funding decisions. By far the largest single investment in research technology at any one of the hospitals was a purchase during the 1954/55 financial year of an ultra-high voltage linear accelerator. The machine alone cost £51,797, and performed radiotherapy cancer treatment. Mr Williams, Head of Department, and Prof Joseph Rotblat persuaded the Executive Committee of the purchase’s necessity for ‘research work and experimentation which would continue to keep them in the forefront of Radiotherapy work in this country’.^77^ This investment was designed to raise prestige, but status and future-focused knowledge were not the only incentives. Bart’s Cancer Committee later reported that the ‘15MeV Linear Accelerator is being brought into use for treatment and clinical research in April 1961, but will continue to be used for part of each day for fundamental research’. The report stated that ‘The research expenses … cannot be separated from those: (i) of its use in treating patients, and (ii) a larger programme of clinical cancer research’. The machine’s research and running costs were divided between the DF, the Ministry of Health, and the British Empire Cancer Campaign (BECC).^78^ While the USH accountants incorporated donations from separate charities like the Yorkshire Council of the British Empire Cancer Research Fund into their endowment, Bart’s administrators seemed to keep the BECC separate from the DF.^79^

Although conducted on a smaller financial scale, the USH’s flagship projects also highlighted from the outset the ambiguity of the border between research and pioneering treatment. Its first large research project was the Sheffield Centre for the Investigation and Treatment of Rheumatism in the early 1950s, financed and managed jointly with the SRHB. The Centre facilitated experimentation and novel treatment, with annual reports boasting of international research links and advertising the use of the cutting-edge drug, cortisone.^80^ The RIE’s Managers similarly used publicity materials to position research and clinical leadership as core to the Infirmary’s identity. For example, their 1960/61 report celebrated a year of ‘marked activity’ in which Dr Michael Woodroof made an ‘outstanding contribution’ to medicine by performing a kidney transplant that ‘captured the imagination of the whole country’. This remarkable achievement was portrayed as a sign that ‘The infirmary is now once again a hive of activity and great work is being done in the fields of clinical medicine in research and in teaching.’^81^ At all three hospitals, research and clinical excellence bled into one another, with charity harnessed to advance medical prestige.

Similar rhetorical use of charity was made by USH’s Governors. Their reports promoted the modern hospital culture that it enabled, in line with Government aspirations for the NHS, declaring that research converged with clinical distinction and thereby established a dynamic medical workforce in the northern city. Their 1953 report commended the Ministry of Health’s recommendation that the NHS ought ‘to foster the research spirit in medicine which is demanded by the highest standards of clinical practice and to facilitate the discovery and encouragement of local talent’.^82^ However, the endowment’s limitations came to dampen this optimism. The Board later argued that Government expectations were exerting increasing demands on endowments as the landscape of medical innovation changed, announcing in 1963 that,

Modern medicine grows increasingly complex and advance cannot be adequate unless resources can be devoted to research. A great teaching hospital has an important role to play in this field and the Board expect to devote an even larger share of their own funds to research in the future.^83^

While this situation created pressure on charitable resources in Sheffield, it presented freedom of experimentation and investment for the more affluent Bart’s and the RIE with its diverse sources of research funding. Nonetheless, the Government did seemingly respond to the USH Governors’ concerns, beginning to issue some Exchequer research grants the following year and thus tacitly acknowledging the border’s impracticality.

## Expenditure categories: routine medical and surgical equipment

All three Boards used charitable funds for medical and surgical items deemed routine, with managers feeling obliged to compensate for Treasury stringency to keep pace with drugs and equipment costs. The RIE’s Board, for example, was unimpressed by its income during the NHS’s early years. Its first report surveyed the Service’s first decade and complained that:

Since the inception of the National Health Service the limitation of funds made available to the Royal Infirmary has necessarily restricted the purchase of Equipment from Exchequer funds, but with the aid of the Board’s Endowment Fund numerous improvements have been financed including the purchase of 1,200 interior spring mattresses … and the installation of bed curtains …^84^

These purchases were depicted as vital for offsetting Exchequer shortfalls in delivering up-to-date services.

Slightly later, our English cases indicated growing reliance on endowments for routine equipment. The USH’s Board was concerned during the early 1960s about the mounting cost of medical supplies in a context of increasingly high-tech interventions, and in 1961/62 spent £6,229 of endowment money on ‘large and expensive items of medical and surgical equipment’, including items for the new Cardio-Thoracic Surgery Unit. It explained that ‘the Fund continues to bear the costs of a number of items which are essential if an up-to-date service is to be provided but which is increasingly difficult to finance from the limited Ministry of Health grant’.^85^ The issue escalated, and the 1969 accounts listed ‘medical equipment’ as a distinct category of endowment expenditure, logging £9,779 (22 per cent) under this heading.

Bart’s DF accounts introduced the heading ‘equipment’ in 1963/64, when £9,215 was spent, rising to £29,982 in 1967/68. In 1967, Bart’s Finance Officer warned that ‘serious consideration would now have to be given to reducing some of the recurrent revenue commitments of the Fund’, which was now meeting sums ‘properly chargeable to the Exchequer revenue account’, including £9,634 spent on ‘Medical and surgical appliances and equipment’.^86^ The financial manager emphasized that there was a border between routine and research equipment, and that crossing it to make good Treasury deficits from the endowment was unsustainable.

Employees at all three hospitals articulated disquiet about these escalating day-to-day costs, concluding that the increasingly technical nature of medicine and the shift towards disposable products simply required more money. For example, in 1959, the RIE’s Pharmaceuticals Department communicated their inability to stem ‘increased spending on disposable face masks, rubber gloves, new surgical instruments, sterile fluids, etc’.^87^ The problem intensified throughout the 1960s. In 1969, representatives of Bart’s met with the Department of Health and Social Security to discuss budgets. Bart’s representatives warned that ‘the expenditure explosion on disposable products made it extremely difficult to hold back spending’ on ‘Normal Medical and Surgical Supplies and Equipment’.^88^

Neither the Government nor the hospitals’ leaders relished deploying endowments to counteract Treasury shortfalls for drugs and equipment. The Government’s favoured solution to rising costs was promoting efficiency. Ministry of Health and Treasury directives emphasized the need for an efficient, organized, and dependable NHS.^89^ As Davies highlights, following the publication in 1956 of the Guillebaud Report, which endorsed the need for substantial expenditure to maintain adequate standards, the Government promoted management techniques and productivity science. To ensure that NHS subventions were spent efficiently, it created the Advisory Council for Management Efficiency in 1959.^90^ All three hospitals collaborated with these campaigns, founding specialized committees to promote drug and equipment economy.^91^

Nonetheless, these efficiency drives did not reduce the costs of routine medical items below Treasury revenue budgets. Boards reluctantly continued to spend voluntary money on such items to avoid diminishing their medical offer. Indeed, the USH’s 1970/71 report listed £5,211 under ‘Contribution to Exchequer Expenditure: Medical and Surgical Equipment’, in a bald statement that charity was compensating for inadequate Treasury allocations.^92^ The theoretical border between state responsibility for routine items and the charitable remit of additional resources remained formally in place at the USH and Bart’s. These BoGs breached it out of a pragmatic need to meet the increasing costliness of modern medical supplies while expressing their distaste for this necessity. The rhetoric of a sharp border was thereby used to lobby for increased Treasury budgets.

## Expenditure categories: patient and staff amenities

Despite Bevan’s protests regarding the social connotations of charity, its persistence allowed administrators to harness voluntarism as a means and language to signal the character of the nationalized hospital. In this vein, amenities and comforts were initially designated obvious domains for endowments. This relatively small use of charity became an outsized public signifier that NHS teaching hospitals had the capacity to look after well-being as well as health. ‘Amenities’ attracted considerable proportions of the English hospitals’ voluntary outgoings in the NHS’s first years, but the category came to represent smaller percentages of outlay (without necessarily seeing reductions in real terms investment) as endowment expenditure diversified over time. Our comparative approach also illuminates how the conceptual integrity of the Bevanite border wavered in practice. Historical wealth inequalities led managers of different institutions to disagree about what was ‘essential’.

Financial analysis shows that, while patient and staff comforts remained an important aspect of hospital philanthropy throughout the period, they attracted a smaller proportion of charitable money than was initially anticipated. The RIE accounts simply report such expenditure under ‘Miscellaneous, Including Amenities’. Between 1957/58 and 1972/73, yearly spending levels fluctuated without a pattern, ranging from £2,002 to £9,080, though this category consistently received the second largest proportion of expenditure, significantly behind ‘Alterations, Furniture And Equipment’. The English accounts provide more detail and distinguish patient from staff amenities. The proportion of USH endowment expenditure dedicated to ‘patients’ welfare and amenities’ saw an unsteady downward trend (see Fig. 2) from 58 per cent in 1950/51 to 12 per cent in 1972/73, although real terms expenditure actually rose slightly. Generally, the proportion of Bart’s DF spent on patient amenities was very small, at between 1 and 3 per cent of outgoings as of 1952/53. While the DF contained most of Bart’s endowments, smaller separate funds existed, some of which were dedicated to patient amenities. USH staff amenities expenditure displayed a less clear pattern, fluctuating between 8 and 39 per cent of endowment outgoings. At Bart’s, staff amenities initially dominated endowment outgoings, accounting for 61 per cent of DF spending in 1949/50. Again, there was a proportionate decrease, although spending held up in real terms.

**Figure 5:**
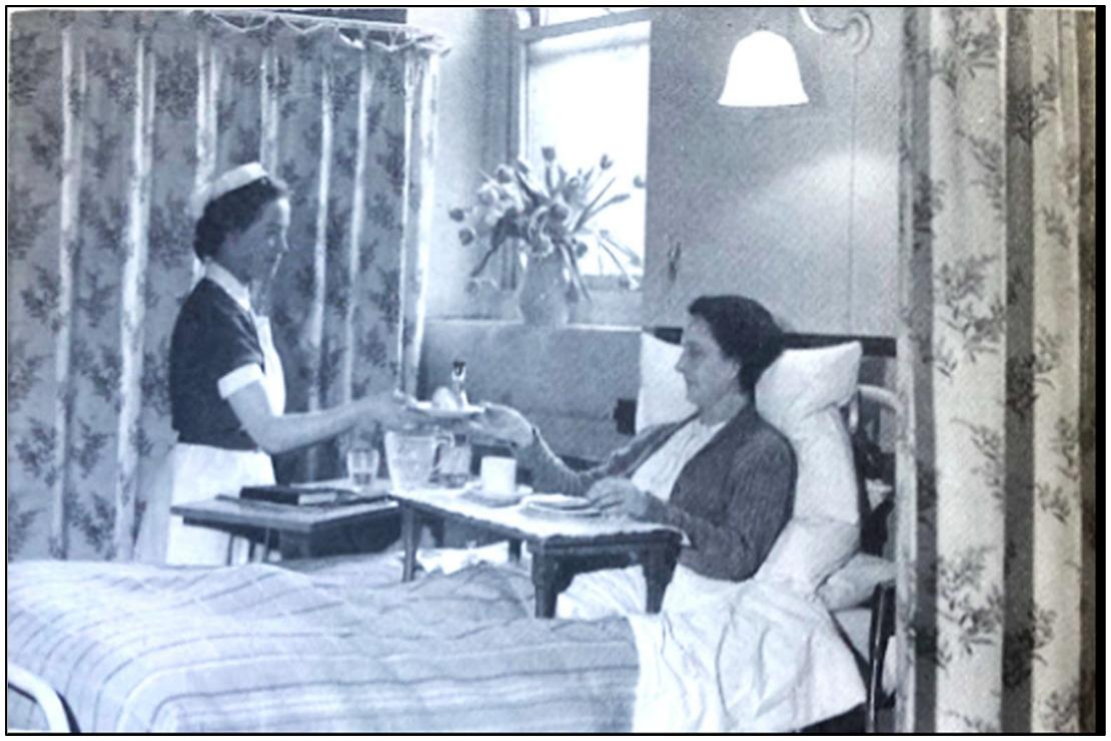
‘The privacy provided by recently installed bed curtains is appreciated by both patients and staff’, *Sheffield AR*, 1952, 14. Sheffield City Archives NHS28/1/2/1/2.

Some patient amenities were uncontroversial, seen as additional comforts at all three hospitals. These included Christmas celebrations, televisions, and library services.^93^ The USH’s and Bart’s largest outgoings on staff amenities were on accommodation, whereas smaller improvements to working life, including flowers and nurses’ prizes, were also provided.^94^ However, the distinction between amenities and essentials proved contestable, and varied with the hospitals’ wealth. As noted, RIE management complained of compensating for Treasury shortfalls during the 1950s by purchasing bed curtains through endowments.^95^ Conversely, in 1952, the Governors of the less affluent USH proudly noted the ‘considerable sums’ of endowment money invested in bed curtains, which were not seen as a basic requirement, but were introduced on an ‘experimental basis’.^96^ Communications about this relatively modest deployment of funds promoted the affective environment that managers of the modern nationalized teaching hospital hoped to achieve. Photographic representation of bed curtains at the USH (Fig. 5) announced that charity lent warmth and humanity, signalled by the floral spray, patterned fabrics, fulsome pillows, meal tray, and tactile postures. Simultaneously, it normalized the expectation of privacy as part of medical interaction, albeit within the ‘communalism’ of the NHS as it transitioned from wartime austerity.^97^

Patient and staff amenities have, over the NHS’s life, stayed firmly within charity’s remit for additional items and services. Abnett, Bowles and Mohan have demonstrated through analysis of accounts from 676 NHS charities in England and Wales between 2015 and 2020 that spending on ‘patients’ welfare and amenities’ remained common and continued to cover items like flowers, newspapers and toys.^98^ However, scholars have built on the historically unstable division between ‘amenity’ and core service to question whether ‘hospital comforts’ should be seen as ‘inessential’ at all. Stewart and Cresswell have highlighted historical attitudes to the border here to argue that—as we were dramatically reminded by the Covid-19 pandemic—‘patient and staff wellbeing should not be left to charitable funding’.^99^ Our analysis further highlights that the Bevanite border drew a questionable distinction between biomedical health and mental well-being.

The conceptual distinction between essential treatment and additional support was particularly porous for ‘convalescence’. In the English institutions, convalescent services were provided by separate philanthropic organizations, reflecting the messy division between medical intervention and social care drawn during the NHS’s early years.^100^ Indeed, the USH’s BoG treated such activity both as a type of medical intervention and a form of philanthropic support. The 1956 report acknowledged how:

The reliance now placed by the hospital almoners on the continuing support of the Talbot Cuff Convalescent Fund, the Sheffield and District Convalescent and Hospital Services Council, the Zachary Merton Charity for Convalescents, the Sheffield Hospitals Sunday Fund … shows the invaluable assistance which these bodies render towards the complete recovery of patients and their return to normal life.^101^

Here, convalescence was deemed integral to recovery, but its separation from acute treatment meant that it acquired a status adjacent to social care, and came to occupy a space that was simultaneously the remit of hospitals, charities, and local authorities, and truly the domain of none of these service-providers. Convalescence was similarly financed at Bart’s through various special funds kept apart from the DF, including the Catherine Marsh Convalescent Home Fund and the Metropolitan Sunday Fund.^102^ In 1964/5 and 1965/66 £2,235 and £2,340, respectively was spent from the Catherine Marsh Fund, which was seemingly the largest pot used for such purposes. While we can see that outgoings on patient amenities were higher than analysing the DF alone suggests, Bart’s Governors still dedicated a much smaller proportion of their considerable free monies to such causes than the USH Governors did from their more modest means. Conversely, the RIE group included two convalescent institutions, Corstorphine House and Beechmount Hospital, that were incorporated into the nationalized hospital.^103^ In 1961, the former was upgraded to Corstorphine Convalescent Hospital, with the costs of this project ‘ … paid from the Board of Management Endowment Fund’ (the early-1960s peak in Fig. 1).^104^ The expanded facility had 112 beds and, though its physical upgrade was charitably financed, the hospital was run using Exchequer budgets.^105^ While convalescence at the RIE was an NHS-funded enterprise in terms of day-to-day staffing and equipment, substantial sums of charitable money had enabled the construction of modern infrastructure for that purpose.

## Expenditure categories: capital

The managers of our teaching hospitals saw capital investment as the Exchequer’s responsibility. However, the Boards quickly began to use their endowments to expand facilities and modernize infrastructure as Treasury stringency became apparent. Each Board tackled localized problems with differing levels of resources, but all presented their actions as pragmatic breaches of a border that ought to have been upheld.

During the 1950s, these hospitals began to harness voluntary reserves for capital projects. Sheffield’s Governors expressed concerns from the outset that the old voluntary hospitals’ buildings were unsuitable for modern healthcare. In 1948, they warned of ‘a large accumulation of “capital works”’.^106^ By the mid-1950s, there had been a consequent upswing in charitable capital expenditure. Between 1956/57 and 1960/61, the Board invested £25,001 of endowment money in a new psychiatric clinic. The Treasury provided about half the project’s cost. An annual report explained that:

In view of the restrictions on capital expenditure from Exchequer sources and bearing in mind the urgent need for this additional accommodation for psychiatric cases from the clinical, teaching and research point of view, the Board has decided to finance the cost of the adaptation partly from its Treasury allocation and partly from the Endowment Fund.^107^

Governors felt that the USH’s lack of psychiatric unit (all treatment had heretofore been provided through the RHB’s Middlewood Mental Hospital) rendered the teaching hospital outmoded.^108^ Creating a new unit was thus presented as a vital act of modernization.

Over the years, charitable capital expenditure at the USH became more routine. By far the greatest such investment occurred during the group’s final years, when the BoG spent the MPA money on the new Hallamshire Hospital. Efforts to develop a new general hospital had begun when leaders of the Royal Infirmary and Royal Hospital had launched the Appeal in 1938. The USH’s Board began planning the project in earnest in the 1950s, and the new hospital was built in stages, with the first department opening in 1961.^109^ Work continued throughout the USH’s remaining lifespan, and it was anticipated that the new Sheffield Area Health Authority (Teaching) would bring the Hallamshire into full use in 1976.^110^ During the USH’s final two years, charitable money (endowment plus MPA) contributed 17 per cent of capital expenditure. Voluntary funds, therefore, significantly contributed to constructing a whole new teaching hospital. The BoG had not been exaggerating the need to replace crumbling buildings: an evacuation of the Royal Infirmary was forced in 1970 after floors were found to be structurally unsafe.^111^ Replacement buildings thus very much came under the banner of essential NHS infrastructure.

Capital expenditure from Bart’s DF far exceeded these levels. Figure 3 shows that Bart’s accountants did not label any of the DF’s outgoings as ‘capital expenditure’ until the early 1950s. The graph then shows a relatively modest capital outlay until 1956/57, after which capital expenditure briefly dominated the DF budget, consuming 82–83 per cent of spending, 1958/9-1960/1, before returning to modest levels in 1963/64, then increasing again from 1967/68.

After a few years, Bart’s Governors became comfortable using their large endowment to expedite projects for which Treasury money was not forthcoming. The Board began its largest charitably funded capital scheme during the mid-1950s, using £699,847 of the DF between 1954/5 and 1963/4 to construct an ‘L-shaped Block’. This sum was enormous compared to anything expended at the USH, and more than double that raised under Sheffield’s MPA. Bart’s Governors wished to complete the block quickly and their charitable wealth gave them the power to do so. Representatives of Bart’s and the Ministry of Health met to negotiate the project’s commencement date, with Bart’s Executive Committee Chair urging ‘that it was of the utmost importance that the L-Shaped Block should be started and completed as soon as possible because only in this way could we hope completely to evacuate Hill End’.^112^ The BoG was happy to use DF money to circumvent any delays resulting from Exchequer limitations, provided that harnessing their endowment would incur no reduction in Treasury allocations. A Ministry of Health circular soon confirmed that ‘building operations carved out by the Hospital of its free monies would not be counted against the Ministry’s capital allocation for building purposes’.^113^ The project thus began. The block was to house multiple clinical specialisms, such as neurosurgery and tuberculosis.^114^

By the end of the Board’s tenure, using DF money for capital projects was routine. Bart’s decline in voluntary capital spending in the mid-1960s was accompanied by an overall reduction in the endowment’s outgoings, and also coincided with increased use of the DF for ‘maintenance of buildings and associated equipment’. Nonetheless, the theoretical border continued to exist in the BoG’s eyes. In 1967, the Executive and Finance Committee’s Clerk had ‘reminded the Ministry of the expenditure from the Endowment Funds in respect of the operating needs of the service’, exerting pressure for a Treasury contribution towards nurses’ accommodation.^115^ Nonetheless, Bart’s Board continued to spend its endowment to enable upgrades and extensions alongside maintenance work.

The RIE Board’s communications regarding capital spending reflected the greater freedom of voluntary expenditure expressed in the HEC’s guidance. The Infirmary’s reports were consistently forthright about a general necessity to harness charitable funds for infrastructure schemes. Unlike the USH’s leaders, Edinburgh’s Board did not publicly justify each charitable project. The latter instead argued broadly for urgent investment in buildings, and occasionally acknowledged voluntary financing of certain developments. Like Sheffield’s Governors, managers warned of aging facilities.^116^ The BoM emphasized a consequent difficulty of fulfilling modern medical expectations amidst Exchequer limitations, complaining in 1959 that:

More could be accomplished if the necessary moneys were forthcoming from the Regional Hospital Board. Quite substantial costs are being met from the Board of Management’s own resources to keep the Royal Infirmary up-to-date with modern progress and new procedures and at the same time improve conditions for patients and staff.^117^

Annual reports consistently depicted the use of charitable money for core hospital infrastructure as a necessary response to these pressures on resources.

The Board continued to stress the need for upgrades even after the Government agreed to plans to replace aged buildings. As at the USH, a decision was made to replace the old Infirmary with a new hospital. Edinburgh’s scheme was officially announced in 1959.^118^ Despite the forthcoming new hospital, the Board felt it ‘imperative that in this interregnum many projects must be put in hand to ensure that this hospital keeps abreast with modern hospital developments’.^119^ The ostensible need to upgrade buildings earmarked for closure was compounded by delays to the Infirmary rebuilding programme. Indeed, shortly before the NHS reorganization, the Board announced their hopes for the first construction phase to begin in 1973.^120^ The BoM thus funded an extensive upgrade of the Accident and Emergency Department, and continued to harness free monies for infrastructure projects throughout the 1960s and early 1970s.^121^

**Figure 6:**
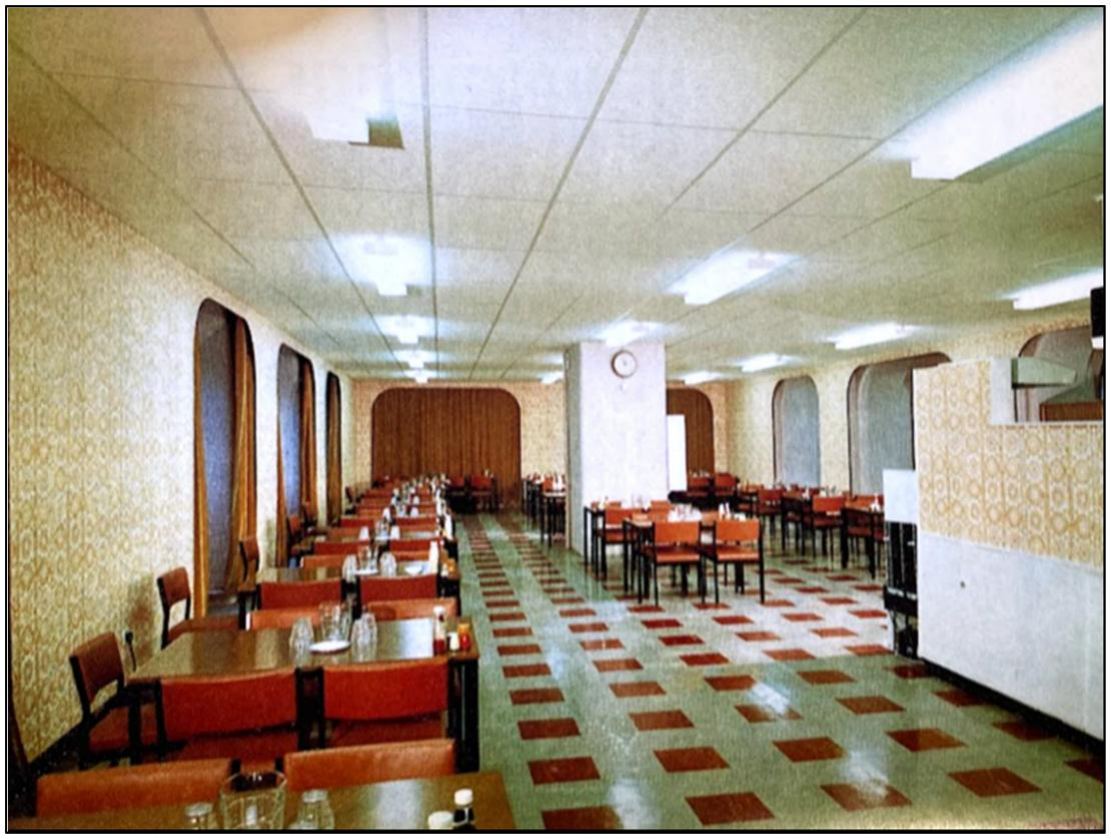
The new dining room, *USH, AR*, 1972: 10. Sheffield City Archives NHS28/1/2/1/5.

The importance of such work to administrators went beyond the pragmatic. Instead, it was shot through with a discourse of ‘modern progress’ both internally and in public representations. We present another image from the 1972 Sheffield Annual Report (Fig. 6) to convey how charitable capacity was deployed to evoke positive feelings for both hospital and NHS. A new dining room is depicted, presenting a vision of post-war modernism conveyed through strong perspective lines, squared furniture, elliptical insets, and geometric floor patterns. The image is from a glossy colour reproduction, and the interior is clothed in what Bates has called the ‘person-centred pastel shades’ of the social democratic welfare state, offset by bolder hues that denote the confident development that charity has facilitated.^122^ Any notion of a meaningful border between essential and non-essential capital spending proved chimeric, as charitable spending underpinned larger claims about the NHS hospital in the ‘decade … of its maturity’.^123^

## Conclusion

The NHS’s founding legislation for hospitals was predicated on a distinction between state-funded core services and charitably-financed non-essentials. We have shown that, while this theoretical division was nominally observed by trustees as the framework for decision-making, a strict border could not be maintained in reality. For research, the boundary between experimentation and cutting-edge treatment quickly blurred, while costly developments in pharmacotherapies led to charitable expenditure on ostensibly routine drugs and equipment. Capital investments in infrastructure soon loomed large in our case-study hospitals, as immediate needs, combined with Exchequer stringency, led the Boards to expedite projects through voluntary reserves. Even where these were spent on amenities for patients and staff, the difference between extras and necessities—from bed curtains to convalescent homes—was subject to interpretation.

A proviso should be entered. Our case study hospitals were drawn from diverse areas of Britain, using different inheritances with which to address localized problems. Generalization from these about patterns and time-trends in expenditure would be problematic. However, our institutional-level findings about the border’s porosity align with the judgments reached by Harris and Cresswell based on central records, and it would be surprising if this general conclusion were overturned. A feature of NHS-style health systems that is now better appreciated is that centralized, taxation-funded structures breed an inherent temptation to state parsimony. Governmental ability to reduce budgets, either due to decisions to prioritize spending elsewhere or from general conservative retrenchment, generates a large space for charity that, with its inherent inequities, cannot be so neatly managed as Bevan had hoped.

Can our findings also contribute to a larger argument about continuities in welfare pluralism across the crucial juncture of 1948? Not really, for the contribution of charitable wealth to the NHS budget was, and remains, too slight. In 2019/20 charitable income (for which estimates vary wildly) comprised just 0.1–1 per cent of most English acute NHS trusts’ incomes.^124^ That said, for select individual hospitals, the charitable reserve could make a major difference to capital spending at key moments, as we have seen for Bart’s. Just as small-scale active volunteering continued into the NHS then, so too did management of assets accumulated in the philanthropic past. Here is an important nuance to narratives of health voluntarism hung only around associations and social movements. It is also a corrective to simplistic ‘moving frontier’ histories, which conceptualize charity’s role post-1945 only as pioneering new forms of social intervention where the state would eventually follow.

Finally, the border remained as a rhetorical device that hospital managers harnessed to lobby for greater funding. They highlighted their enforced reliance on charitable monies for vital infrastructure projects and routine medical supplies, reminding the Government of its self-proclaimed responsibility for these expenses. They also deployed the benefits of charitable spending to burnish the image of their hospitals and to inculcate affection for the NHS. Presenting their publics with visions of technical expertise alongside humane care, Boards made charity integral to the modernizing health service, legitimizing its place in the welfare state.

## Notes

1 See M. Powell, ‘Introduction: The Mixed Economy of Welfare and the Social Division of Welfare’, in M. Powell, ed., *Understanding the Mixed Economy of Welfare* (Bristol: Policy Press, 2007), 1–22.

2 M. Gorsky, ‘British Health Policy, 1919-1948’, in A. Mold and V. Berridge, eds, *Lessons from the History of British Health Policy* (London: The British Academy, 2023), 52–69.

3
*Inter alia* B. Abel-Smith, *The Hospitals 1800–1948: A Study in Social Administration in England and Wales* (London: Heinemann, 1964); B. Doyle, *The Politics of Hospital Provision in Early 20th-Century Britain* (London: Pickering and Chatto, 2014).

4 Parliamentary Debates (Commons), 422, 30 Apr. 1946, 44, 46–48.

5 W. Beveridge, *Voluntary Action A Report on Methods of Social Advance* (London: G. Allen & Unwin, 1948), 8; British Library of Political and Economic Science, London, BHCSA 3/12, Aneurin Bevan, address to the conference of the British Hospitals Contributory Schemes Association Folkestone, 1 Oct. 1948.

6 Abel-Smith, *The Hospitals*; exemplary case studies: A. Borsay, *Medicine and Charity in Georgian Bath: A Social History of the General Infirmary, c.1739-1830*, (Aldershot: Ashgate, 1999); M.E. Fissell, *Patients, Power and Poor in Eighteenth Century Bristol*, (Cambridge: Cambridge University Press, 1991).

7 B. Harris and R. Cresswell, ‘The Legacy of Voluntarism: Charitable Funding in the early NHS’, *Economic History Review*, 77 (2024), 557.

8 M. Gorsky, J. Mohan and M. Powell, ‘British Voluntary Hospitals, 1871-1938: The Geography of Provision and Utilization’, *Journal of Historical Geography*, 25 (1999), 463–82.

9 M. Gorsky, J. Mohan and M. Powell, ‘The Financial Health of Voluntary Hospitals in Inter-war Britain’, *Economic History Review*, lv (2002), 533–57.

10 Harris and Cresswell, ‘Legacy’, 562–63; LHSA, Edinburgh, RIE, LHB2/13/11, Hospital Endowments Commission, ‘Board of Management for the Royal Infirmary of Edinburgh and Associated Hospitals Endowments Scheme Memorandum’ (1954), 1.

11 J. Mohan and M. Gorsky, *Don’t Look Back? Voluntary and Charitable Finance of Hospitals in Britain, Past and Present* (London: Office of Health Economics/ Association of Chartered Certified Accountants, 2001), 91–5; Harris and Cresswell, ‘Legacy’, 554–83.

12 C. Webster*, The Health Services Since the War. Volume 1, Problems Of Health Care: The National Health Service Before 1957* (London: HMSO, 1998), 104, 264–74.

13 ‘National Health Service Appeals for funds etc.’

14
*National Health Service Act, 1946, 9 & 10 Geo. 6 Ch 81*, Section 7 paras 2, 4, 6–7.

15 The National Archives: Public Record Office, Kew, BG (48) 23.a, ‘National Health Service Appeals for funds etc.’ 1948.

16 B. Harris, *The Origins of the British Welfare State: Society, State and Social Welfare in England and Wales, 1800-1945* (Basingstoke: Palgrave Macmillan, 2004).

17 G. Finlayson, *Citizen, State and Social Welfare in Britain* (Oxford: Clarendon, 1994), 287–8.

18 N. Barr, *The Economics of the Welfare State* (Oxford: Oxford University Press, 1998), 78–88; L. Salamon, *Partners in Public Service: Government-Nonprofit Relations in the Modern Welfare State* (London: Johns Hopkins University Press, 1995).

19 M. Gorsky, ‘“Voluntarism” in English health and welfare: visions of history’, in S. Lucey and V. Crossman eds, *Health Care, Voluntarism and Regionalism in Ireland and Britain 1850-1950* (London: Institute of Historical Research, 2015), 31–60; See C. Braithwaite, *The Voluntary Citizen: an Enquiry into the Place of Philanthropy in the Community* (London, Methuen & Co., 1938); E. Macadam, *The New Philanthropy: A Study in the Relations Between the Statutory and Voluntary Social Services* (London: G. Allen & Unwin, 1934); A.F.C. Bourdillon, ‘Introductory’, in idem ed., *Voluntary Social Services: Their Place in the Modern State* (London: Methuen & Co, 1945), 2.

20 R.M. Titmuss, *Problems of Social Policy* (London: HMSO, 1950).

21 Gorsky, Mohan and Powell, ‘Financial health of voluntary hospitals’; M. Gorsky and J. Mohan, ‘London’s Voluntary Hospitals in the Interwar Period: Growth, Transformation, or Crisis?’, *Nonprofit and Voluntary Sector Quarterly*, 30 (2001), 247–75.

22 Doyle, *Politics of Hospital Provision*; N. Hayes and B. Doyle, ‘Eggs, Rags and Whist Drives: Popular Munificence and the Development of Provincial Medical Voluntarism between the Wars’, *Bulletin of the Institute of Historical Research* 86 (2013), 712–40; G. Gosling, ‘“Open the other Eye”: Payment, Civic Duty and Hospital Contributory Schemes in Bristol, c.1927–1948’, *Medical History* 54 (2010), 475–94. G. Gosling, *Payment and Philanthropy in British healthcare, 1918-48* (Manchester: Manchester University Press, 2017).

23 M. Hilton et al., *A Historical Guide to NGOs in Britain: Charities, Civil Society and the Voluntary Sector Since 1945* (Basingstoke: Palgrave Macmillan, 2012); Gorsky, ‘“Voluntarism” in English Health and Welfare’.

24 V. Berridge and A. Mold, ‘Professionalisation, New Social Movements and Voluntary Action in the Nineteen-Sixties and Nineteen-Seventies’, in M. Hilton and J. McKay, eds, *The Ages of Voluntarism: how we got to the Big Society* (Oxford: Oxford University Press, 2011), 114–34.

25 A. Seaton, ‘The Gospel of Wealth and the National Health: The Rockefeller Foundation and Social Medicine in Britain’s NHS, 1945–60’, *Bulletin of the History of Medicine*, 94 (2020), 91–124.

26 Ellen Stewart, *How Britain Loves the NHS: Practices of Care and Contestation* (Bristol: Policy Press, 2023); G. Millward, ‘“Its Many Workers and Subscribers Feel that their Services can Still be of Benefit”: Hospital Leagues of Friends in the English West Midlands, c.1948–98’, *Social History of Medicine*, 36 (2023), 433–55; R. Piggott, ‘Hospital Sunday and the New National Health Service: An End to the “voluntary spirit” in England?’, *Studies in Church History*, 58 (2022), 372–93.

27 J. Crane, ‘‘Loving’ the National Health Service: Social Surveys and Activist Feelings’, in J. Crane and J. Hand eds., *Posters, Protests, and Prescriptions: Cultural histories of the National Health Service in Britain* (Manchester: Manchester University Press, 2022), 79–102; A. Whitecross, ‘The Cultural Significance of Space and Place in the National Health Service’, in Ibid., 177–98.

28 A. Seaton*, Our NHS: A History of Britain’s Best Loved Institution* (New Haven: Yale University Press, 2023); V.L. Bates, ‘Cold White of Day: White, Colour, and Materiality in the Twentieth-century British Hospital’, *Twentieth Century British History*, 34, (2023), 1–37.

29 Harris and Cresswell, ‘Legacy’.

30 R. Meakin, *Charity in the NHS: Policy and Practice* (Bristol: Jordan Publishing, 1998).

31 Mohan and Gorsky, *Don’t Look Back?*; M. Gorsky, and A. Arnold-Forster, *Witness Seminar: NHS Hospital Charity Fundraising Since the 1980s* (London: London School of Hygiene and Tropical Medicine, 2024), ISBN: 978-0-9957874-7-6.

32 J. Bowles, D. Clifford and J. Mohan, ‘The Place of Charity in a Public Health Service: Inequality and Persistence in Charitable Support for NHS Trusts in England’, *Social Science & Medicine*, 322 (2023), 1–10.

33 M. Lattimer and K. Holly, *Charity and NHS reform: A Report into the Changing Role of Charitable Funds in The NHS* (London: Directory of Social Change, 1992); M. Lattimer, K. Holly, and S. Kovats, *The Gift of Health: The NHS, Charity and the Mixed Economy of Healthcare* (London: Directory of Social Change, 1996).

34 H. Abnett, J. Bowles and J. Mohan, ‘The Role of Charitable Funding in the Provision of Public Services: The Case of the English and Welsh National Health Service’, *Policy & Politics*, 51, (2023), 362–84; Bowles, Clifford and Mohan, ‘The place of charity’, 1–10.

35 E. Stewart, R. Cresswell and C. Möller, ‘National Charitable Fundraising for the NHS, 1948–2023’, *Social Policy & Administration*, 58 (2024), 967.

36 Harris and Cresswell, ‘Legacy’, 559–60.

37 Lothian Health Services Archive (LHSA), Edinburgh, RIE, LHB2/3/1-16, ‘The Royal Edinburgh Infirmary and Associated Hospitals Report’ (Edinburgh, 1948-58-1972/73) Hereafter RIE Annual Reports (ARs); RIE AR, 1948-58, 20; Harris and Cresswell, ‘Legacy’, 8.

38 K. Waddington, *Medical Education at St Bartholomew’s Hospital 1123-1995* (Woodbridge: Boydell Press, 2003), 1–10.

39 Barts Health Archives (BHA), https://www.calmview.co.uk/BartsHealth/CalmView/Record.aspx?src=CalmView.Catalog&id=SBHA, 25/02/25.

40 BHA and Museums, ‘Research Guide: NHS Hospital Administration in The City and East London’, (2022), 3.

41 NHS Lothian, https://org.nhslothian.scot/aboutus/royal-infirmary-of-edinburgh-history/, 25/2/25.

42 RIE AR, 1958/59, 5.

43 Harris and Cresswell, ‘Legacy’, 562–63; ‘Scottish Regional Hospital Boards’, *The Lancet*, 2,459 (1947), 698.

44 S. Sturdy, ‘The Political Economy of Scientific Medicine: Science, Education and the Transformation of Medical Practice in Sheffield, 1890-1922’*, Medical History*, 36 (1992), 139; Sheffield City Archives (SCA), Sheffield, The USH and Related Organisations, *The United Sheffield Hospitals Report of the Board of Governors 1948/49-1973/74*, NHS28/1/2/1/1-6. Hereafter USH ARs; USH AR, 1964, 14; USH AR, 1972, 45. S. Isaac, ‘Sheffield Steel and Early Physiotherapy’, Royal College of Surgeons of England (2017), https://www.rcseng.ac.uk/library-and-publications/library/blog/sheffield-steel-and-early-physiotherapy/, accessed 16 March 2026.

45 Sheffield Regional Hospital Board, NHS17, SCA, Sheffield.

46 SCA, https://www2.calmview.co.uk/SheffieldArchives/CalmView/Record.aspx?src=CalmView.Catalog&id=NHS28&pos=1, accessed 16 March 2026.

47 W. Jackson, ‘“The Collector Will Call”: Controlling Philanthropy through the Annual Reports of the Royal Infirmary of Edinburgh, 1837–1856’, *Accounting History Review*, 22 (2012), 52–55.

48 USH AR, 1948/1949, 12.

49 RIE AR, 1948–58, 20.

50 BHA, London, Records of St Bartholomew’s Hospital (hereafter Records of Bart’s), SBHB/HA7/1/6, Minutes Executive and Finance Committee 1957–59, 44–45.

51 BHA, London, Records of Bart’s, SBHB/HB/48/2-21, Annual Accounts and Financial Statements for the Year Ended 31 Mar 1951-Year Ended 31 Mar. 1973.

52 BHA, London, Records of Bart’s, SBHB/HB/18/3/4-24, Discretionary Fund Annual Accounts and Balance Sheet for the Year Ended 31 Mar 1949–Year Ended 31 Mar. 1969. Hereafter Bart’s DF Accounts.

53 BHA, London, Records of Bart’s, SBHB/HA/7/1/1-11, Minutes Executive and Finance Committee 1948–74. Hereafter Bart’s Executive and Finance Committee; BHA, London, Records of Bart’s, SBHB/HA/7/29/1, Research Committee Minutes, 1953–60; BHA, London, Records of Bart’s, SBHB/HA/7/26/1, Joint Research Board Committee 1961–68.

54 Calculated from the British Voluntary Hospitals database, see Martin Gorsky, John Mohan, ‘Uses of Yearbooks: The Voluntary Hospitals Database’, *Social History of Medicine*, 24, 2, 2011, 478–83.

55 USH AR, 1948/49, 11-29; we exclude from this total, and calculation, the ‘MPA’.

56 ‘Memorandum’, 1–7.

57 BHA, London, Records of Bart’s, SBHVB/F/7/2, Metropolitan Hospital Sunday Fund monies for St Bartholomew’s Hospital, accounts for the year ended 30 Jun 1951; ‘Metropolitan Hospital Sunday Fund’, *British Medical Journal*, 2 (1877), 703–04.

58 USH AR, 1953/53, 22; Annual Accounts and Financial Statements for the Year Ended 31 Mar. 1951.

59 Harris and Cresswell, ‘Legacy’, 560–62.

60 Calculated using the data available in ‘Memorandum’, 1–7. Total value of endowment: £1,324,128 (1948 prices); £316,123 redistributed (1954 prices) =24.6%.

61 Harris and Cresswell, ‘Legacy’, 563.

62 Bowles, Clifford and Mohan, ‘The place of charity’, 1.

63 RIE AR, 1962/63, 75; 1948–58, 58.

64 RIE AR, 1948–58, 20.

65 RIE AR, 1967/68, 20.

66 RIE AR, 1972/73, 76.

67 In 1973 the USH’s endowment totalled £389,143, and the RIE endowment plus BoM Fund totalled £354,167. USH AR, 1973, 36; RIE AR, 1972/3, 102–05.

68 USH AR, 1948/49, 21.

69 Bart’s Executive and Finance Committee, 6/1/49, 79, 2/4/49, 108.

70 ‘Memorandum’, 2.

71 ‘Memorandum’, 4.

72 USH AR, 1948/49, 12.

73 Parliamentary Debates (Commons), 422, 30 April 1946, 63; Parliamentary Debates (Commons), 425, 22 July 1946, 1,793-4.

74 USH AR, 1964, 18.

75 RIE AR 1970/71, 56.

76 RIE AR, 1958/59, 21.

77 Bart’s Executive and Finance Committee, 16/2/50, 305.

78 Joint Research Board, 2nd Meeting, 12/4/61.

79 USH AR, 1958, 43.

80 USH AR, 1951, 28.

81 RIE AR, 1960/61, 37.

82 USH AR, 1953, 17.

83 USH AR, 1962/63, 16.

84 RIE AR, 1948-58, 21-22.

85 USH AR, 1961, 19; USH AR, 1962, 16.

86 Bart’s Executive and Finance Committee, 27/6/67, 3–4.

87 RIE AR, 1958/59, 27.

88 Bart’s Executive and Finance Committee, 28/4/69, 1.

89 G. O’Hara, *From Dreams to Disillusionment Economic and Social Planning in 1960s Britain* (Basingstoke: Palgrave Macmillan, 2006), 167–204.

90 S.M. Davies, ‘Promoting Productivity in the National Health Service, 1950 to 1966’, *Contemporary British History*, 31 (2017), 47–68.

91 RIE AR, 1959/60, 27; Bart’ Executive and Finance Committee, 5/10/61, 300-301; USH AR, 1970, 18.

92 USH AR, 1971, 39.

93 Bart’s DF Accounts, 1963/64-1968/60; RIE ARs, 1948-58-1972/73; USH ARs, 1951–1973.

94 Bart’s DF Accounts, 1963/64-1968/69; RIE AR, 1972-73, 41; USH ARs, 1951–73.

95 RIE AR, 1948-58, 21–22.

96 USH AR, 1952, 14.

97 Seaton, *Our NHS*, 65-6, 76–80.

98 Abnett, Bowles and Mohan, ‘The role of charitable funding’, 374.

99 E. Stewart and R. Cresswell, ‘All the Trimmings: Patient and Staff Wellbeing should not be Left to Charitable Funding’, *BMJ* (2023), 383.

100 USH AR, 1959, 40.

101 USH AR, 1956, 14.

102 BHA, London, Records of Bart’s, SBHVB/F/8/, Audited accounts of the Voluntary Board for the year ended 31 Dec 1966, along with those of the ‘special funds’ managed by St Bartholomew’s Hospital.

103 RIE AR, 1958/59, 5.

104 RIE AR, 1960/61, 21.

105 RIE AR, 1961/62, 30.

106 USH AR, 1948/49, 23.

107 USH AR, 1955, 22.

108 USH AR, 1955, 19.

109 USH AR, 1969, 46.

110 USH AR, 1974, 21.

111 USH AR, 1970, 20.

112 Bart’s Executive and Finance Committee, 4/3/54, 69.

113 Bart’s Executive and Finance Committee, 18/3/53, 78.

114 Bart’s Executive and Finance Committee, 21/4/55, 206.

115 RIBA pix, https://www.ribapix.com/gloucester-house-nurses-home-extension-st-bartholomews-hospital-bartholomew-close-city-of-london-a-nurses-room-with-built-in-wash-basin_riba50369, 25 Feb. 2025; Bart’s Executive and Finance Committee, 23/2/67, 9–10.

116 RIE AR, 1948-58, 23.

117 RIE AR, 1958/59, 24.

118 RIE AR, 1958/59, 20.

119 RIE AR, 1959/60, 33.

120 RIE AR, 1971/2, 23.

121 LHSA, Edinburgh, RIE, LHB21632, ‘The New Accident and Emergency Department’, *The Consultative*, 1 (1967), 36–37.

122 Bates, ‘Cold white of day’, 37.

123 Seaton, *Our NHS*, 59.

124 Bowles, Clifford and Mohan, ‘The place of charity’, 5.

