# Correction to the following two papers

**DOI:** 10.1093/tcbh/hwag017

**Published:** 2026-05-18

This is a correction to: Hannah Blythe, Martin Gorsky, ‘Free From Doubt’? The border between charity and the state in the British National Health Service, 1948–74, *Modern British History*, Volume 37, Issue 2, 2026, https://doi.org/10.1093/tcbh/hwag005 and Ben Jackson, Tom Nairn and Scottish Politics, *Modern British History*, Volume 37, Issue 2, 2026, https://doi.org/10.1093/tcbh/hwag011

The original publication of these papers were made with erroneous issue values.

The articles are now published to the correct issue and the issue values are now emended in the article to read: Hannah Blythe, Martin Gorsky, ‘Free From Doubt’? The border between charity and the state in the British National Health Service, 1948–74, *Modern British History*, Volume 37, Issue 1, 2026, https://doi.org/10.1093/tcbh/hwag005 and Ben Jackson, Tom Nairn and Scottish Politics, *Modern British History*, Volume 37, Issue 1, 2026, https://doi.org/10.1093/tcbh/hwag011.

